# Short inverted repeats contribute to localized mutability in human somatic cells

**DOI:** 10.1093/nar/gkx731

**Published:** 2017-08-22

**Authors:** Xueqing Zou, Sandro Morganella, Dominik Glodzik, Helen Davies, Yilin Li, Michael R. Stratton, Serena Nik-Zainal

**Affiliations:** 1 Wellcome Trust Sanger Institute, Hinxton, Cambridge CB10 1SA, UK; 2 Department of Biosciences, University of Helsinki, FI-00014 Helsinki, Finland; 3 East Anglian Medical Genetics Service, Cambridge University Hospitals NHS Foundation Trust, Cambridge CB2 9NB, UK

## Abstract

Selected repetitive sequences termed short inverted repeats (SIRs) have the propensity to form secondary DNA structures called hairpins. SIRs comprise palindromic arm sequences separated by short spacer sequences that form the hairpin stem and loop respectively. Here, we show that SIRs confer an increase in localized mutability in breast cancer, which is domain-dependent with the greatest mutability observed within spacer sequences (∼1.35-fold above background). Mutability is influenced by factors that increase the likelihood of formation of hairpins such as loop lengths (of 4–5 bp) and stem lengths (of 7–15 bp). Increased mutability is an intrinsic property of SIRs as evidenced by how almost all mutational processes demonstrate a higher rate of mutagenesis of spacer sequences. We further identified 88 spacer sequences showing enrichment from 1.8- to 90-fold of local mutability distributed across 283 sites in the genome that intriguingly, can be used to inform the biological status of a tumor.

## INTRODUCTION

**Figure 1.**
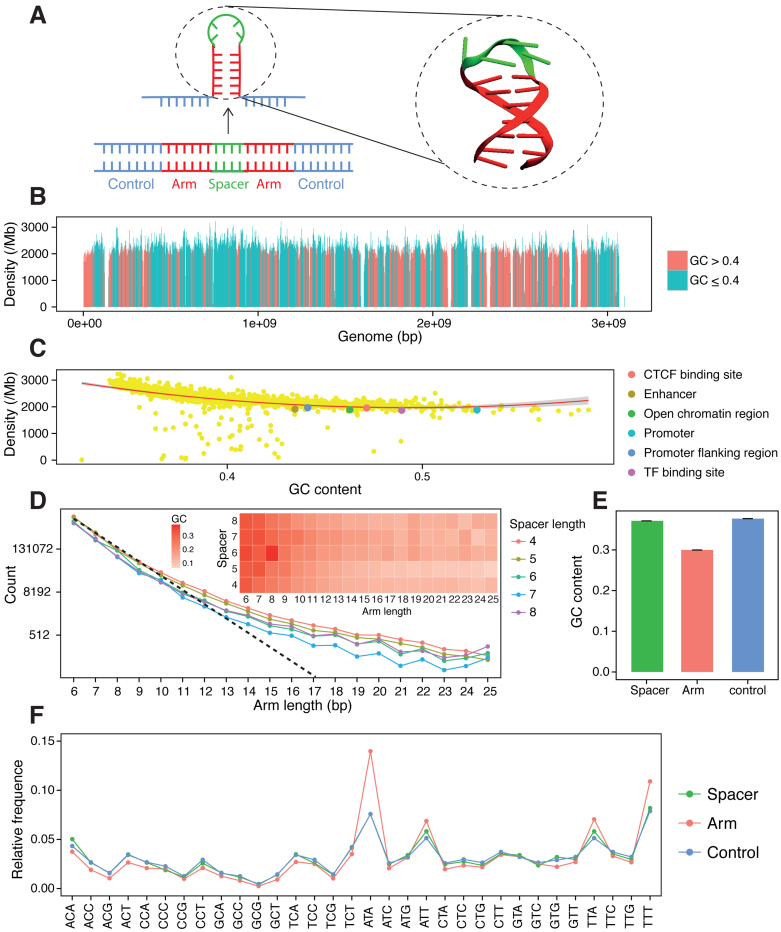
Genomic properties of short inverted repeats (SIRs). (**A**) A schematic illustration of hairpin formation froms an inverted repeat (IR), where spacer forms the loop (green) and palindromic arms forming the stem (red). A total of 100 bp in upstream and 100 bp downstream of each SIR are used as control (blue). A 3D structure of hairpin is shown. (**B**) Density of SIR in genome. Each bar represents the density of SIRs in a size of 2 Mb bin. Red shows the GC content of the bin is >0.4 and cyan shows the GC content of the bin is ≤0.4. (**C**) The relationship between density of SIRs and local GC content. Each yellow dot represents a bin from (A). A quadratic line was fitted to the data, showing the increase of local GC content reduces the density of SIRs when GC <0.5. Densities of SIRs from six regulatory element regions (red: CTCF-binding sit; dark golden: enhancer; green: open chromatin region (OCR); cyan: promoter; blue: promoter flanking region; purple: TF-binding site) fit to modeled line, indicating that distribution of SIRs is not dependent on regulatory element regions, but rather the GC content of them. (**D**) Number of SIRs with various spacer length and arm length. The dashed line shows the expected decrease of number of SIRs. The GC level of SIRs with different arm length and spacer length is shown in right inset. (**E**) GC content differs in spacer (green), palindromic arm (red) and control regions (blue), where GC_spacer_ = GC_control_ > GC_arm_. (**F**) Relative frequency of 32 trinucleotides in spacer, arm and control regions.

Beyond the linear arrangement of primary nucleic acid sequence, human DNA can form higher order physical configurations. Apart from assuming the customary right-handed double helix, selected repetitive sequences have the potential to adopt alternative secondary structures called non-B DNA conformations (1–5). A particular type of repetitive sequence called inverted repeat (IR) comprises two reverse complementary sequences (or palindromes), separated by several nucleotides, termed spacer sequences. When DNA is single-stranded DNA (ssDNA), palindromic arms can transiently hybridize to form a stem structure while the intervening spacer sequence forms a loop—in all, creating a secondary structure called a DNA hairpin (6,7) (Figure 1A). Two hairpins on opposing ssDNA strands can lead to a cruciform structure (8,9). Hairpin and cruciform formations require DNA to be single-stranded, no matter how transiently and this could occur during physiological processes such as transcription or replication (10–13).

Critically, hairpin structures contribute to mutagenicity (13–16). For example, by examining germline sequences from the 1000 Genomes Project, non-B DNA regions were found to have a higher density of polymorphic variants than control regions (17). Experimentally, IRs were also reported to initiate genomic amplification by inducing a type of intrachromosomal rearrangement, a so-called fold-back inversion (18–20). Two groups further reported that IRs are enriched at translocation and deletion breakpoints in yeast cells (21) and mammalian cells (21,22), suggesting that IRs could induce double-strand breaks. Recently, in order to detect novel non-coding driver mutations in human cancers, statistical methods were used to pinpoint loci that are mutated at a higher frequency than expected (23). This attempt identified recurrent mutagenesis at the promoter of the *PLEKHS1* gene. This was corroborated in a separate experiment examining 560 whole breast cancer genomes (Supplementary Table S1) (24). Interestingly, the recurrent mutations at this promoter occurred at two specific sites within the spacer sequence of an IR (the underlined nucleotides of GAAC/GTTC). Intriguingly, additional sites of recurrent mutagenesis were observed with identical spacer sequences in the breast cancers (24). This led us to ask whether DNA hairpin structures, could generally influence local mutability in human somatic cells.

We thus systematically characterized all SIRs across the genome and explored relationships with mutability of all classes of mutations. Prior studies have indicated that multiple factors contribute toward thermodynamic stability of a hairpin structure including stem and loop lengths, sequence composition and cellular milieu (e.g. salt concentration) (25–31). IRs with loop lengths of ∼4–5 nts and IRs with arm lengths of ≥7 bp have been shown to confer optimal stability for hairpin formation (32,33). However, the stability of hairpin structures reaches a plateau at about 25 bp (34). We thus focused on SIRs with spacer lengths between 4 and 8 bp and palindromic arm lengths between 6 and 25 bp, because these are most likely to form stable hairpin structures *in vivo*.

## MATERIALS AND METHODS

### Dataset

A previously published dataset of somatic mutations including substitutions, indels and rearrangements of 560 breast cancers (24) was used in this study. Somatic mutations are caused by multiple mutational processes. Each comprises DNA damage and DNA repair components, and generates a distinct pattern called a mutational signature (35–37). The final mutational profile of each cancer is a combination of all the mutational signatures that have been operative through the lifetime of the cancer patient. By applying mathematical methods, mutational signatures can be extracted and quantified in each cancer (38). Previous studies have identified over 30 mutational signatures across 40 types of cancers (36). In the 560 breast cancer dataset, 12 mutational signatures were previously extracted from substitutions (24), which are signature 1 (associated with deamination of 5-methylcytosine), signatures 2 and 13 (associated with the activity of the AID/APOBEC family of cytidine deaminases; AID: activation-induced cytidine deaminase; APOBEC: apolipoprotein B mRNA editing enzyme, catalytic polypeptide), signature 3 (homologous recombination deficiency), signature 5 (unknown aetiology), signatures 6, 20 and 26 (associated with defective DNA mismatch repair) and signatures 8, 17, 18 and 30 (all of unknown aetiology) (24). The 560 samples were grouped into five classes by performing hierarchical clustering on mutational signatures of each sample: APOBEC samples (Signature 2/13), APOBEC+MSI samples (Signature 2/13 and 6/20/26), microsatellite instability (MSI) samples (Signature 6/20/26), BRCA (*BRCA1/BRCA2*-null) samples (Signature 3) and other samples. A total of 174 exomes of bladder and cervix cancers were also examined (Supplementary Table S1 regarding data).

### Characterization of SIRs in the genome

The GRCh37/hg19 human reference genome assembly was used to search for SIRs. The IR searching program was written in python. We first used biopython package to search for all IR sequences which contain a 4–8 bp spacer and 6–25 bp flanking palindromic arms. We obtained 100 putative groups of SIRs (5 spacer sizes * 20 arm sizes). Duplicated SIR sequences (that showed up in different groups), were reduced to be represented only once into the most stably predicted hairpin, which is when the loop is as short as possible but ≥4 bp (steric constraints) and the arm is as long as possible (34). For example, IR sequence AGGCTAGCTGGCTAGCCT can be categorized into 3 groups: 4 bp-spacer and 7 bp-arm, 6 bp-spacer and 6 bp-arm, as well as 8 bp-spacer and 5 bp-arm. We confine it to one category (4 bp-spacer and 7 bp-arm) as it is predicted to be the most stable hairpin conformation. We removed all IRs with pure AT spacers (see Figure S1 in Supplementary Methods). At low complexity sequence regions, several SIRs may overlap with each other. When a sequence could theoretically contain multiple overlapping SIRs, we used only the longest possible predicted SIR.

### Somatic variants in SIRs

For each SIR, the number of substitutions and indels from 560 cancer samples were interrogated in spacer sequences, arms and in control sequences (100 bp flanking each SIR). In general, there is a lower density of rearrangement breakpoints in human tumors, thus in this analysis, the control sequences for analysis of rearrangement breakpoints relative to SIRs involved 1000 bp flanking sequences.

### Exploring variation in mutability of SIRs

The null hypothesis states that there is no difference in density of substitutions in the spacer sequences and in control sequences. Thus, having identified all mutated SIRs, we first verified whether each SIR had an elevated mutation density when compared to flanking control sequences (Supplementary Figure S2). *P*-values were calculated using a binomial test, with multiple hypothesis testing correction, to evaluate the degree of significance. SIRs with an adjusted *P*-value ≤ 0.01 were labeled as ‘highly mutated SIRs’. To ensure that the elevated mutation density of the spacer sequence was not simply a general property of the particular sequences of the spacers, we compared mutabilities of the spacer sequences which were flanked by palindromic arms (and were thus SIRs) to identical sequences that were not flanked by palindromes. Binomial tests were performed to calculate *P*-values (corrected for multiple hypothesis testing) to identify spacer sequences within SIRs that were significantly highly mutated (termed SIR hotspots).

Out of 6 622 303 SIRs, there are 54 998 SIRs with substitutions found in spacers, in which 552 are identified as highly mutated SIRs. These 552 SIRs were distributed across 283 different locations in the genome, as some locations have multiple SIRs overlapping (these locations are usually GC = 0 and we chose the longest SIR as a representative for the location). Among the highly mutated SIRs, some spacer sequences were seen recurrently. In all, 88 unique spacer sequences are particularly highly mutated, see Supplementary Methods.

### Genomic features

We also examined relationships between SIRs and a variety of features of genomic architecture to see if mutability was influenced by regulatory elements or by the mechanics of cellular physiological processes such as replication and transcription. Reference coordinates for replication timing domains and regulatory features were described in previous publications (39,40). Mutability of SIRs were systematically explored across all of these features.

### Statistics

All statistical tests were performed in R. In particular, binom.test (41) was used for binomial test to determine if mutation density in spacer deviates from mutation density in control sequences. The *P*-value of the test can be obtained by using binom.test(*N*_muts_spacer_, *L*_spacer_*560, *d*_muts_control_, alter = ‘greater’), where *N*_muts_spacer_ is the number of mutations found in spacers, *L*_spacer_ is the length of the spacer, 560 is the number of genomes in the dataset and *d*_muts_control_ is the mutation density in control sequences. t.test was used for*t*-test; Since we examined a large group of SIRs at the same time, p.adjust(*P*-values,‘BH’) was used for multiple testing correction to reduce false positive calls in our analysis (42). lm was used to perform linear regression. Biostrings was used to compute reverse-complement of DNA sequences (43,44). All plots were generated by ggplot2 (45).

## RESULTS

### Systematic characterization of short inverted repeats (SIRs) in the human genome

A total of 6 622 303 SIRs were identified showing variable densities of between 2000 and 3000 SIRs per Mb throughout the reference human genome. This uneven distribution is associated with genomic GC content (Figure 1B), where SIRs occur more frequently in AT-rich regions, described best by a quadratic function (Figure 1C). The enrichment of SIR in AT-rich regions has also been observed in a previous study (46). Distributions of SIRs at regulatory element regions (Supplementary Figure S3 and Figure 1C) and replicating timing domains (Supplementary Figure S4) are also in keeping with their GC content. SIRs with shorter arm lengths tend to show higher GC content than SIRs with longer palindromic arms (inset in Figure 1D and Supplementary Figure S5). The expected likelihood of detecting an SIR should decrease exponentially as arm length increases (dotted line Figure 1D). However, a departure from this expected trend was observed, relating to longer arm lengths being particularly enriched at AT-rich regions of lower complexity (see Supplementary Results).

### SIRs are more mutable than their surrounding sequences

We also considered control sequences in our analyses defining these as a window of 100 nts flanking each SIR. GC content of palindromic arms is significantly lower than that of spacer or control sequences (Figure 1E and Supplementary Figure S6). Taking trinucleotide sequence context into account (Figure 1F), spacer sequences have greater similarity in sequence composition to control sequences (cosine similarity = 0.997) than arm sequences (cosine similarity = 0.949) which have more ATA, ATT, TTA and TTT trinucleotides. The high degree of likeness between spacer and flanking sequences make the latter a reassuring choice as controls.

#### Increased SIR mutability is domain dependent

**Figure 2.**
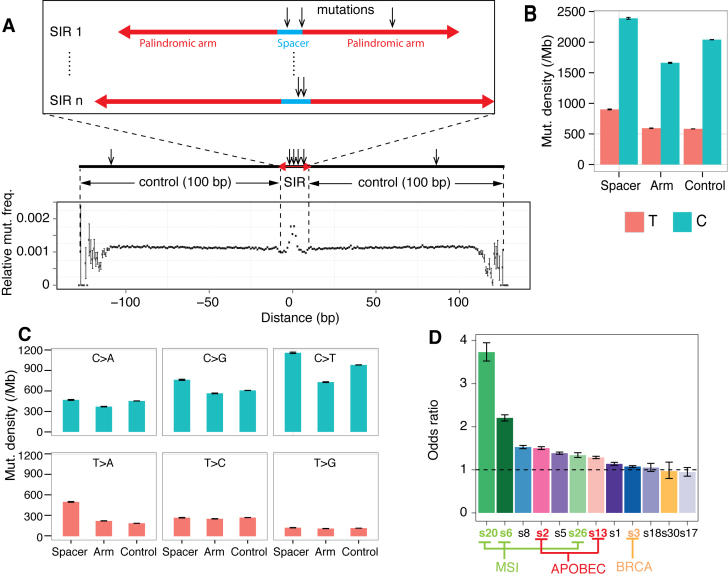
Substitutions in SIRs. (**A**) The relationship between the likelihood of finding a mutation and the distance of the mutation to the center of an SIR. (**B**) Comparison of mutation density between spacer, arm and control. Substitutions are referred to by the pyrimidine of the mutated base pair. (**C**) Mutation density of six substitution types. (**D**) The odds ratio (OR) of mutation density of spacer to control for 12 mutational signatures found in breast cancers is shown. Signature 2 and 13, attributed to the activity of the AID/APOBEC family of cytidine deaminases (APOBEC, red). Signature 3 is strongly associated with BRCA1/BRCA2 mutations (BRCA, orange). Signatures 6, 20 and 26 are found in tumors with mismatch repair deficiency, causing microsatellite instability (MSI, light green).

Having defined the reference set of SIRs and control sequences, relationships with base substitution mutagenesis were examined. Substitution densities exhibit a distinct peak toward the center of SIRs (Figure 2A), particularly at spacer sequences (∼1.35 times higher than background). C:G and T:A mutations occur more frequently in spacer sequences than in control (Figure 2B) or arm sequences (*P* < 2.2 × 10^−16^ for both C and T). Divided into six classes of base substitutions (Figure 2C), SIR spacer sequences showed the highest densities for C > G, C > T and T > A (*P* < 2.2 × 10^−16^ for all three substitution classes) mutations compared to control sequences, while arm sequences showed lower mutation densities for C > A, C > G, C > T, T > C, T > G. Using 96 classes of base substitutions (that take the flanking sequence context for each altered base into account), spacer sequences are enriched for (but not exclusively due to) C > T transitions at a TCN context (OR > 1) and for T > A transversions at ATT, TTA and TTT (OR > 5) compared with control sequences (Supplementary Figure S7), indicating that elevated substitution mutation densities in spacer sequences are influenced by sequence context.

#### Increased mutability of SIRs is an intrinsic property of SIRs

Mutational signatures are the patterns of mutagenesis that are left by the activities of DNA damage and DNA repair pathways that have been operative in human cells (36). These mutational signatures have previously been shown to demonstrate sequence context dependence (36). We therefore asked whether the elevated mutation density of spacer sequences was driven by specific mutational processes. Intriguingly, we find that most of the substitution signatures demonstrate a higher level of mutagenesis within spacer sequences than arm or control sequences (OR > 1), as shown in Figure 2D. This would suggest that the increased mutability observed at spacers is an intrinsic property of the loop region within DNA hairpin structures, irrespective of mutational process present in each tumor.

However, tumors with mismatch repair deficiency (MMRd), tumors that show a high level of activity of the APOBEC cytidine deaminases and tumors with high Signatures 5 and 8 (both of unknown aetiology), demonstrate the most dramatic fold increase of spacer mutability compared to control sequences (Figure 2D and Supplementary Figure S8). Thus, additional factors must contribute to mutability of spacer sequences given the variation in effect size observed between mutational processes.

**Figure 3.**
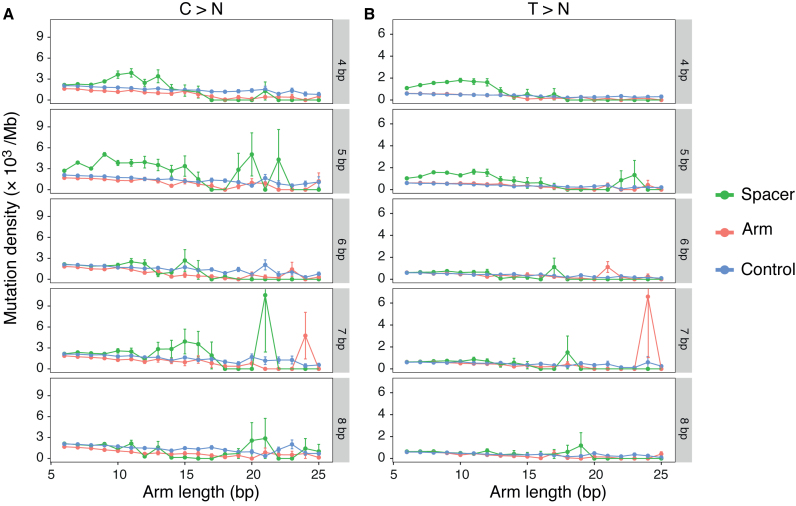
Mutability of SIRs is influenced by physical properties associated with DNA hairpin formation. Here the influence of spacer length and arm length on mutation density of SIRs is shown. SIRs are separated into 100 groups according to the spacer (4–8 bp) and arm (6–25 bp) lengths. We calculated the mean mutation density of substitutions for each SIR group. The standard deviations of the means are represented by error bars. C>N mutation rates (**A**) and T>N mutation rates (**B**) of spacer (green), arm (red) and control (blue) in SIRs with spacer length (range from 4 to 8 bp) and arm length (range from 6 to 25 bp). SIRs with spacer length 4–5 bp and arm length 7–15 bp show increase of mutation density in spacer.

If formation of a hairpin structure confers increased local mutability, physical characteristics that affect thermodynamic stability of these structures could influence overall mutagenesis (25). Indeed, we further observed that elevated spacer mutability was restricted to SIRs with spacer lengths of 4–5 bp and arm lengths of 7–15 bp (Figure 3) more than SIRs with arm lengths ≥16 bp. This latter observation may seem counterintuitive theoretically, longer arm lengths would be associated with more stable hairpin formation. However, the likelihood of hairpin formation is also influenced by GC contents: arms with greater GC content are more stable (34). In exploring the general properties of SIRs (Figure 1D), we showed that SIRs with shorter arms tended to have higher GC content, thus, this might account for the observation of elevated mutabilities at those particular arm lengths of 7–15 bp. These biophysical properties are thus additional factors that increase the likelihood of hairpin formation, inspiring localized mutagenesis in human genomes.

### Increased SIR mutability is not uniform

**Figure 4.**
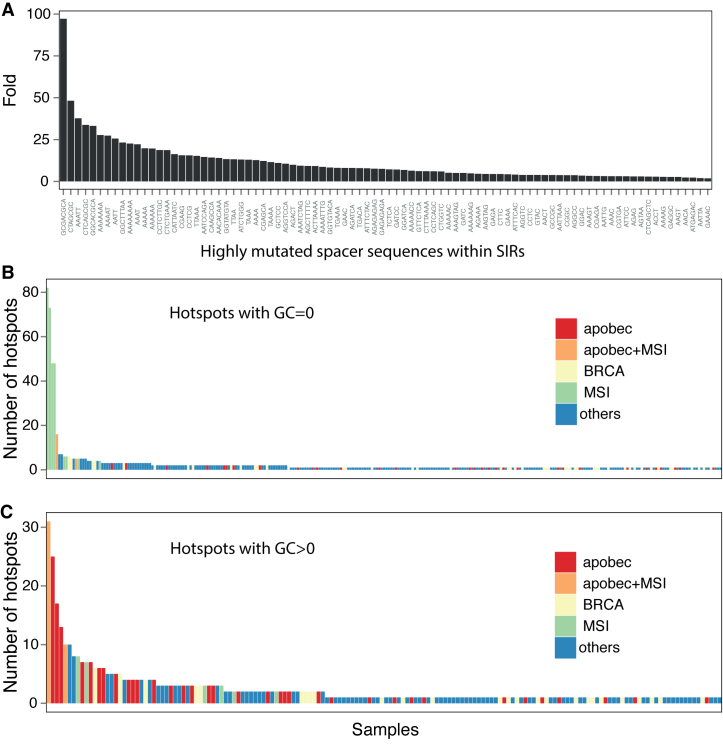
A subset of SIRs are mutation hotspots. (**A**) The fold difference of mutability for spacer sequences within SIRs and for identical sequences that are not within SIRs (i.e. not flanked by palindromic sequences). (**B**) Number of mutated SIR hotspots per sample for hotspots with GC = 0 (**C**) Number of mutated SIR hotspots per sample for hotspots with GC > 0. Samples are also colored by their predominant mutational signature phenotype (APOBEC, red; APOBEC+MSI, orange; BRCA, yellow; MSI, light green; Others, blue).

Although the elevated mutability of spacers is due to the collective effects of SIRs, the contribution of each SIR to mutability is not equal. To identify SIRs that could have higher mutabilities, we compared the mutability of spacer sequences within SIRs to identical sequences that are not flanked by palindromes. We found that 88 spacer sequences have an increased likelihood of mutagenesis (*q*-value ≤ 0.01) when nestled within an SIR (termed highly mutated spacers). Indeed, 31 of these spacers are more than 10-fold more mutable than similar sequences that are not within IRs (Supplementary Table S2 and Figure 4A).

A highly mutated spacer sequence could have different palindromic arms as has been described previously (24). The highly mutated spacer GAAC/GTTC was identified as recurrently mutated at 10 different genomic locations with different palindromic arms at each locus (e.g. chr11:10331381–10331384 palindrome CCTTGGCT/AGCCAAGG; chr 6:142706206–142706209 palindrome CTCTTTGTAT/ATACAAAGAG). We found that these 88 highly mutated spacers are distributed across 283 recurrently mutated SIR sites that we term SIR hotspots (see ‘Materials and Methods’ section; Supplementary Tables S2 and S3).

SIR hotspots can be used as markers of biological status of a breast tumor. Of these, 160/283 (57%) sites were comprised of only A and T nucleotides. Samples found to be mutated at 16 or more of these sites show MSI with at least 34% of the mutations associated with signatures of defective DNA mismatch repair (Signatures 6, 20, 26) (Figure 4B and Supplementary Table S4). The remaining 123 sites were informative for samples with APOBEC (Signatures 2 and 13) activity. Samples that were mutated at seven or more of these sites had evidence of substantial APOBEC mutagenesis, frequently exceeding a third of total mutational burden per sample (Figure 4C and Supplementary Table S4).

### Relationship between SIRs and genomic architecture

**Figure 5.**
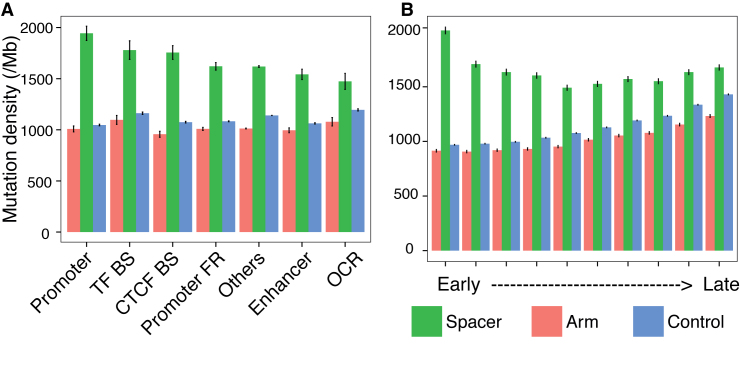
SIR mutagenesis in regulatory elements and replication timing domains. (**A**) Mutation densities of SIR spacers, arms and controls in regulatory element regions, including promoters, transcriptional factor binding sites (TF BS), CTCF binding sites (CTCF BS), promoter flaking regions (promoter FR), others (other regions excluding these six regulatory element regions), enhancers and OCR; (**B**) Mutation densities of SIR spacers, arms and controls in replication timing regions which are divided into deciles.

To identify other factors that could influence the variable mutability of SIRs, we explored whether SIRs in different regulatory features and across early through to late replication timing domains demonstrated differing levels of mutability between SIRs. Interestingly, there were observable differences noted between different regulatory elements and this demonstrated domain dependence. In particular, arm and control sequences appear to have similar mutation densities between 950 and 1100 per Mb and 1000 and 1150 per Mb respectively (Figure 5A) across all regulatory elements examined and other regions (excluding these six regulatory element regions). By contrast, spacer sequences, although markedly and consistently elevated when compared to arm and sequences for all regulatory elements, demonstrate a greater variation in mutation densities (between 1400 and 2000 per Mb). The highest levels of spacer mutability are observed at particular regulatory elements associated with being poised or in an open chromatic state. This includes promoters followed by transcription-factor binding sites, more so than other regulatory elements (Figure 5A). Furthermore, across replication timing domains, spacer mutability is at its highest in the earliest domain of the G2/S phase of the cell cycle (Figure 5B). This is in contrast to mutabilities of control or arm sequences (47) that demonstrate a gradual increase of mutability going from early-to-late domains, in-keeping with observations of substitution density in cancers in general. Thus, mutability of SIRs, particularly of spacer sequences, appear to be increased by being within promoters and transcription-factor binding sites and being in earlier replication timing domains. This could be because the likelihood of formation of hairpins by SIRs is distinct in different regulatory elements and is strongest in early replicating regions because there is more or a longer availability of ssDNA in these regions.

### Relationships between indels and rearrangements with SIRs

Hairpin formation has also been implicated in causing small (1–50 bp) insertions/deletions (indels) during replication (13). Therefore, the effects of SIR on indels and rearrangements were also investigated. It was observed that indels are enriched at spacer sequences by almost 2-fold when compared to control sequences. In contrast to substitutions and indels, the density of breakpoints is only very slightly higher in both SIR spacers and arms than in controls (Supplementary Results).

## DISCUSSION

SIRs appear to contribute toward elevation of mutation densities, but in a highly localized way. This observation is not restricted to breast cancer and is replicated in an analysis involving 136 bladder and 38 cervical cancers as well (Supplementary Figures S9 and 10). Further analyses are required to explore SIRs across all tumor types—particularly those with very different mutational signatures, for example malignant melanomas that have an enormous influence from external sources such as ultraviolet light.

Our analyses show that the increased mutability is specifically focused on spacer sequences that correspond to the loop domain of hairpin structures. The increased mutability occurs for most mutational processes though is particularly augmented in tumors with MMRd, with high APOBEC activity and/or with high levels of Signatures 5 and 8. One explanation is that the likelihood of hairpin formation is the same in all tumors but signatures that have a higher mutational load cause greater mutability of spacers. However, this hypothesis is less likely. First, although an increased mutation rate is often associated with APOBEC activity or MMRd, tumors with deficiency of another repair pathway, homologous recombination repair, also have relatively high mutational loads but do not display as marked a propensity for mutating spacer sequences. Second, Signatures 5 and 8 are not associated with excessive mutational burden and therefore, this reasoning cannot explain the increased mutability of hairpin structures associated with these mutational signatures.

It has been suggested from experimental systems, that the degree of mutability of SIR is related to the likelihood of formation of a secondary structure. Perhaps there exist other pathophysiological qualities associated with tumors that have MMRd, APOBEC activity or Signatures 5 and 8, which increase the likelihood of hairpin formation in these tumors and thus mutagenesis at these spacer sequences. Specifically, an increased availability of single-stranded DNA (ssDNA) during replication or transcription—ssDNA being a prerequisite for hairpin formation. For example, replication has been linked both experimentally and analytically with APOBEC-related mutagenesis (40,48,49). Additionally, both Signatures 5 and 8 are typified by transcriptional strand bias suggesting that transcription could influence hairpin formation and thus spacer mutability for these signatures. There are also other cellular processes that could lead to the formation of ssDNA–long regions of ssDNA accumulate during break-induced replication (BIR) (50–52) and/or microhomology mediated BIR (MMBIR) (53).

Thus, certain biological abnormalities perhaps relating to dysregulated replication, transcription and replicative repair, are likely to increase the availability of ssDNA resulting in an increased propensity for hairpin formation, thus influencing localized mutation rates.

We finally identify SIR mutation hotspots that could be used to report whether a cancer has MMRd- or APOBEC-related activity. These SIR hotspots are specific though not particularly sensitive at reporting these biological statuses. Nevertheless, they could be used as a cheap and quick surrogate marker of these mutational processes should it be necessary to perform limited sequencing on small volumes of DNA in the future. Using simply primary genomic sequences, the propensity to form secondary structures in the human genome that contributes toward mutability, can be exploited to predict tumor biology.

## Supplementary Material

Supplementary Data
