# Supplementary material for: Short inverted repeats contribute to localized mutability in human somatic cells
Source: Nucleic Acids Res. 2017 Aug 22;45(19):11213–21. doi: 10.1093/nar/gkx731 (PMC5737083; doi:10.1093/nar/gkx731)
Supplement: Supplementary Data [file gkx731_supp.zip › nar-01254-i-2017-File007.docx]

**Supplementary Information for**

**Short inverted repeats contribute to localized mutability in human somatic cells**

Xueqing Zou, Sandro Morganella, Dominik Glodzik, Helen Davies, Yilin Li, Michael R. Stratton and Serena Nik-Zainal

**Contents**

**Supplementary Methods**  2

Clean SIR dataset 2

**Supplementary Figures 3 – 10** 4-11

**Supplementary Table 1-4 Captions**  12

**Supplementary Results** 12

Relationship between GC content of SIRs and local GC content 12

GC content and arm length affect IR distribution 13

Recurrent spacer and arm sequences in SIR hotspots 14

Indels in SIRs 14

Rearrangements in SIRs 19

**Supplementary References** 21

**Supplementary Methods**

**Clean SIR dataset.** Many sequences in the genome satisfy the definition of an inverted repeat in which two palindromic sequences are separated by a spacer sequence (Fig. 1a). On deeper inspection, we find that low complexity regions such as AT repetitive sequences could fit this criterion very easily. In the present study, we first removed predicted SIRs that comprised purely of AT repeats. Second, we removed predicted SIRs that had spacers that comprised purely of AT repeats. This helped to greatly reduce the number of sequences that are not true SIRs. Fig. S1 shows the number of SIRs before (a) and after (b and c) removing predicted SIRs as described above.


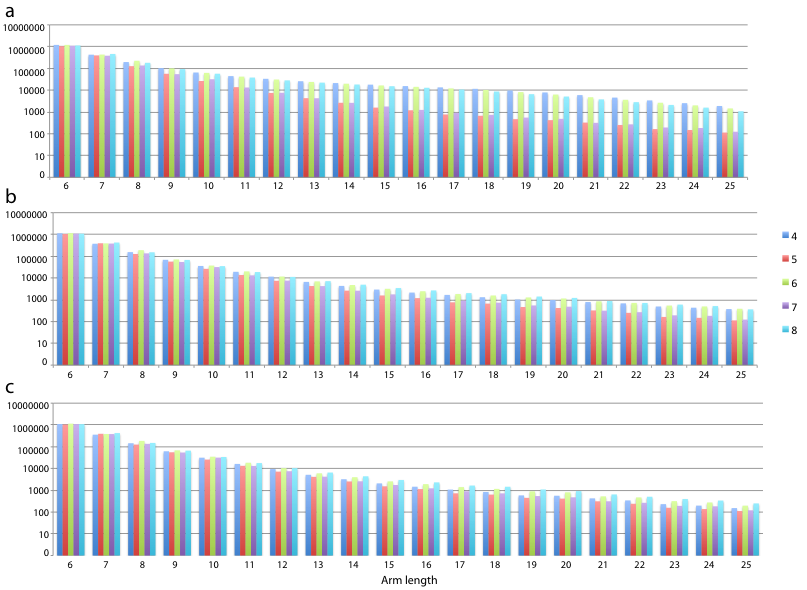


Figure S1. The number of SIRs with arm length >= 6bp and spacer length between 4-8 bp are identified in the genome. (a) All predicted SIRs including those comprising purely of AT repeats are shown. (b) Predicted SIRs comprising purely AT repeats removed. (c) SIRs with purely AT repeats in spacers removed. When pure AT repeats were included in SIR dataset, there were consistently more predicted SIRs with arm lengths of 4, 6, and 8 than with arm lengths of 5 and 7 which is an artefactual effect. This difference was reduced when predicted SIRs that comprised purely of AT repeat sequences or had spacers that were purely AT repeats, were removed.


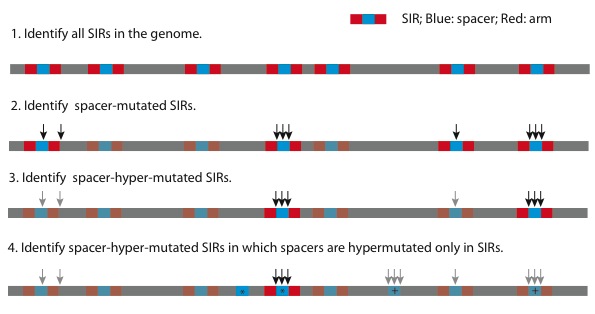


Figure S2. Schematic illustration of four steps to identify SIR mutation hotspots. First, a clean dataset of SIRs with arm >= 6bp and spacer between 4-8 bp in the reference genome was obtained (as described in Fig. S1). Grey line represents the genome; SIRs are highlighted on Grey line. Second, SIRs containing substitutions in their spacers were identified, as highlighted in Fig. S2, step 2. Each black arrow represents a substitution. Third, SIRs with an increased density of mutations (p-value = 0.01) when compared to flanking control sequences were identified and highlighted, step 3. Fourth, spacer sequences that were particularly hyper-mutated were identified, by comparing spacer sequences present within an IR and identical sequences that were not embedded within an IR. As an example, in step 4, a spacer sequence labeled by “*” shows an elevated mutation density only when it is flanked by palindromic sequence and is within an SIR. By contrast, spacer sequence labeled by “+” shows an increased mutation density whether or not it is embedded within an SIR (faded in Fig. S2, step 4). A total of 88 such spacer sequences were identified. These spacer sequences could have different palindromic arms in different parts of the genome. These 88 hypermutated spacers were distributed across 283 SIRs termed SIR mutation hotspots.

**Supplementary Figures**

Figure S3. SIRs are not over-represented at regulatory elements such as CTCF binding sites, enhancers, open chromatin regions, promoters, promoter-flanking regions or transcription factor binding, in-keeping with the higher GC content of these regulatory elements. The red dashed line indicates the average SIR density in the genome.

Figure S4. SIR density increases from early to late replicating time domains as GC content decreases. The red dashed line indicates the average SIR density in the genome.

Figure S5. The GC content of SIR with different arm length. (a) Distribution of GC content of SIR in each group. (b) The mean value of GC content of SIR.

Figure S6. GC content differences between spacer, arm and control sequences of SIRs with different arm length and spacer length.


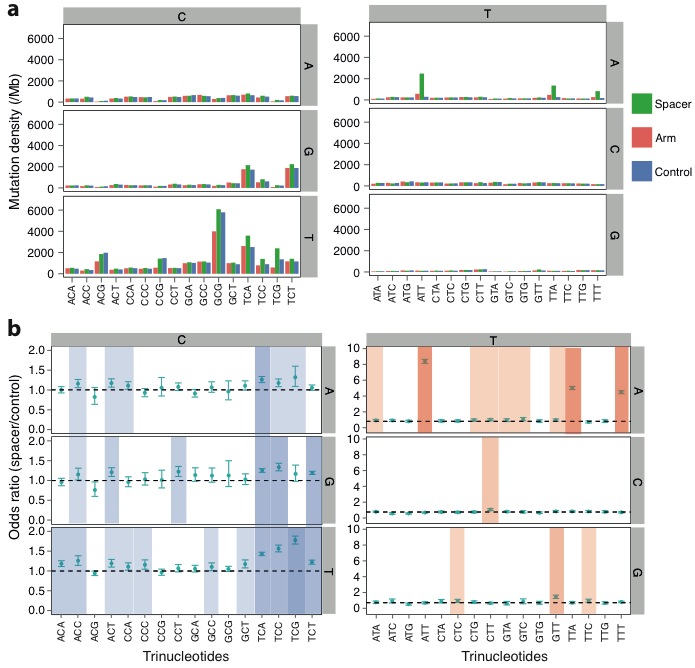


Figure S7. Mutability of 96 substitution types in spacer, arm and control. (a) Comparison of Mutation rate of 96 substitution types among spacer (green), arm (red) and control (blue). (b) Odds ratio between mutation rate in spacer and mutation rate in control. The substitution types with the lower level of 95% confidence interval > 1 were highlighted by blue (C mutations) or orange (T mutations). The darker the color, the higher the odds ratio.

Figure S8. (a) Odds ratio between mutation rate in spacer and mutation rate in control for 560 breast cancer samples. Samples are ordered by the lower boundary of the 95% confidence interval, in decreasing order. Samples are also colored by their predominant mutational signature phenotype (APOBEC, red; BRCA, orange; MSI, light green; Others, blue). Samples on the left of the red dashed line have lower level of 95% CI > 1, while samples on the right have lower level of 95% CI < 1. (b) The percentage of substitution in the spacer versus the total number of substitution in each sample. The inset shows the distribution of spacer substitution in different phenotype samples.

Figure S9. Mutation densities of spacer, arm and control in exomes of 136 bladder cancers. (a) The overall mutation density. (b) GC mutation density. (c) AT mutation density. (d) Mutation density of SIRs in regulatory elements. (e) Mutation density of SIRs in replication timing regions.

Figure S10. Mutation densities of spacer, arm and control in exomes of 38 cervix cancers. (a) The overall mutation density. (b) GC mutation density. (c) AT mutation density. (d) Mutation density of SIRs in regulatory elements. (e) Mutation density of SIRs in replication timing regions.

**Supplementary Table Captions**

**Table S1.** Detailed information about samples examined in the present study, including 560 breast cancers, 136 bladder cancers and 38 cervix cancers.

**Table S2.** Detailed information about 88 spacer sequences showing increased local mutability observed in 560 breast cancers.

**Table S3.** Detailed information about 283 SIR mutation hotspots observed in 560 breast cancers.

**Table S4.** Mutational signature and mutated SIR hotspots information for each breast cancers. Sheet 1 shows the number of SIR hotspots with GC = 0 observed in each sample; Sheet 2 shows the number of SIR hotspots with GC > 0 observed in each sample.

**Supplementary Results**

**Relationship between GC content of SIRs and local GC content.** The GC content of SIRs has a linear relationship with local GC content, as shown in Fig. S11. This linear positive correlation shows, as expected, that the SIRs identified in regions with low GC content are likely to have low GC content as well.

**
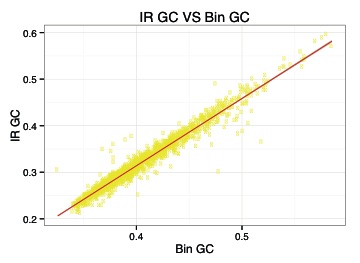
**

Figure S11. GC content of SIRs shows linear relationship with the GC content of local region.

**GC content and arm length affect IR distribution.** SIRs were classified into 100 groups according to their spacer length and arm length ($L_{arm}$). With a given $L_{arm}$, the probability of detecting the reverse complimentary sequence is ${0.25}^{L_{arm}}$. Hence, as $L_{arm}$increases, the likelihood of finding a SIR is expected to decrease exponentially (by base 4), as indicated by the dashed line in (Fig. 1*d*). Instead, we find that the decrease in the number of predicted SIR reduces with the increase of $L_{arm}$(Fig. 1*d*). This is likely to be due to the depletion of GC content and concurrent reduction in sequence complexity for SIRs with longer arm lengths (right inset in Fig. 1*d*). As an example, the probability to find a reverse complimentary sequence of a 6 bp sequence in GC = 0 region (containing only A and T) is 0.5^6 = 0.015625, whilst in GC = 0.5 region (equally containing A, T, G and C) the probability is 0.25^6 = 0.00024, which is much smaller than GC = 0 region. Hence, regions with low sequence complexity are more likely to have higher SIR densities than regions with more mixed nucleotides, as observed in Fig. 1*c*.

Indeed, the percentage of SIRs with low GC content, e.g., GC<0.3, increases with the increase of arm length (Figure S12). Low complexity sequence regions are more likely to meet the algorithmic criteria for finding predicted SIRs, and therefore, the number of SIR with long arms (>=15bp) does not reduce as expected, but rather achieves to a constant number.

Figure S12. The distribution of SIRs with different GC content. Red, green and blue show the percentages of SIRs with GC content = 0, 0< GC content < 30%, and GC content>=30%, respectively.

**Recurrent spacer and arm sequences in SIR hotspots.** Of the cohort of 283 SIR mutation hotspots, here are SIRs (spacer and arm motifs) that recur through the genome. Three SIR sequences occurred multiple times (Table S5). An SIR with a spacer sequence of AGACT and arm sequence GGGAAAGGG is found at five different genomic locations - three are located in CTCF binding sites. An SIR with a GATC spacer sequence flanked by the arm sequence CCTCCTGGT occurs twice, and an SIR with spacer sequence TCTCA with arm sequence, GACAGAG occurs three times.

Table S5. SIR mutation hotspots with recurrent spacer (core) sequences. Recurrent arm sequences flanking around the same spacer (core) sequence are highlighted in yellow.

**Indels in SIRs.** Prior studies have suggested that hairpins may cause insertions and/or deletions (indels) during replication^1,2^. Here, we calculated the mutation densities of indels in SIR spacer, arm and control sequences and found that indels are also enriched in spacers: the indel density in a spacer is almost twice of that in a control (Fig. S13*a*). Fig. S13*b* compares mutation densities in SIR spacer, arm and control sequences according to the 5 subtypes of indels (complex indels, microhomology(mh)-mediated deletions, repeat-mediated deletions, other deletions (which are not mh-mediated and repeat-mediated deletions) and insertions). All classes of indels show elevated mutation densities in spacer regions apart from microhomology-mediated deletions. The reason for this is unclear. However, microhomology-mediated deletions are generally considered to be biologically different in origin relative to other indels. Unlike other indels, these are believed to originate as double-strand breaks and are associated with cancers that have abnormalities of homologous recombination repair deficiency. Thus, it is possible that this underpins the differrent observed behaviour when compared to other classes of indels. We also examined indel density in SIRs with different spacer lengths and arm lengths. Interestingly, repeat-mediated deletions and insertions show high indel density for SIRs with small (4-5 bp) spacer and short (*<* 15 bp) arms (Fig. S13*c-g*), an observation that is very similar to substitutions. The GC content also affects the indel density of SIR. As shown in Fig. S13*h-l*, repeat-mediated deletions, other deletions and insertions are enriched in spacers of SIR with low GC content.

The size of indels indeed affects indel densities in spacer, arm and control sequences. As shown in Fig. S14, when the size of indels increases to 3 bp, the differences of indel density in spacers, arm and control sequences become smaller. Furthermore, we also examined the relationships between the size of indels and the structural features, e.g., the length of spacers and arms, of SIRs. Consistent with Fig. S14, larger indels (>=3 bp) do not show clear difference in spacer and control sequences in all groups. For small size of indels (1-2 bp), the elevated indel densities are seen in SIRs with arm length < 15 bp.

Figure S13. Indel in SIRs. (a) Indel density in spacer (green), arm (red) and control (blue) sequences. (b) Comparison of indel density in five indel types. Complex, microhomology-mediated deletions, repeat-mediated deletions, other deletions, insertion. (c)-(g) Indel rate of SIRS with different spacer length and arm length, separated by five indel types. (h)-(l) Indel rate of SIRs with different GC content, separated by five indel types.

Figure S14. Comparison of densities of indels with different sizes in spacer, arm and control sequences of SIRs. (a) 1 bp insertions; (b) 2 bp insertions; (c) >= 3bp insertions; (d) 1 bp deletions; (e) 2 bp deletions; (f) >= 3 bp deletions.

Figure S15. Comparison of densities of indels with different sizes in IRs. IRs are separated into different groups according to spacer (4-8 bp) and arm lengthes (6 - 25bp). (a) 1 bp insertions; (b) 2 bp insertions; (c) >= 3bp insertions; (d) 1 bp deletions; (e) 2 bp deletions; (f) >= 3 bp deletions.

Influences of regulatory elements, replication strands and replication timing on the distribution of SIR indels were also studied (Fig. S16). The results show that the indel densities were elevated differently in different regulatory element regions. In promoters, indels show highest spacer densities, whilst in CTCF binding sites, SIR spacers indel density is lowest (for substitutions, the lowest elevated mutation density of spacer is in open chromatin regions), see Fig. S16a. Distributions of indels are almost the same in lagging strand and leading strand (Fig.S16b). As to replication timing regions, the densities of indels in arms and controls increase gradually from early to late replicating regions, and indel densities in spacers first decrease and then increase, a behaviour that is similar to that observed for substitutions (Fig. S16c).

Figure S16. Indel densities of SIR spacers, arms and controls in (a) regulatory element regions, including promoters, transcriptional factor binding sites (TF BS), CTCF binding sites (CTCF BS), promoter flaking regions (promoter FR), others, enhancers and open chromatin regions (OCR); (b) replicative strands and (c) replication timing regions. According to DNA replication time data, ten replication regions were identified, from left to right: early to late.

**Rearrangements in SIRs.** The association between SIRs and rearrangements was also investigated. Unlike substitutions and indels, the distribution of rearrangement breakpoints is relatively even across SIR and control regions. The density of breakpoints is slightly higher in SIR spacers and arms than in controls (Fig. S17*a*). By calculating breakpoint densities for each type of rearrangements (deletions, inversions, tandem duplications and translocations), we found that deletion breakpoints were more enriched in SIR spacers and arms than in controls; for inversions and translocations, the breakpoint densities are almost the same in spacers, arms and controls; for tandem duplications, the breakpoint densities in arms are slightly higher than in spacers and controls (Fig. S17*b*). The distribution of breakpoints in regulatory elements, replicative strands and replication timing regions also varies. However, due to limited power, the differences are not clear. One notable thing is that in replication timing regions, the density of breakpoints in SIR spacers decrease from early replication region to late, which follows the same trend of breakpoint densities of arms and controls (Fig. S18).

Figure S17. Breakpoint density in spacer, arm and control. (b) Comparison of breakpoint density in four rearrangement types.

Figure S18. Influence of three genomic features (regulatory elements, replicative strands and replication timing regions) on breakpoint density of SIR spacer, arm and control sequences.

**Supplementary References**

1. Bikard, D., Loot, C., Baharoglu, Z. & Mazel, D. Folded DNA in Action: Hairpin Formation and Biological Functions in Prokaryotes. *Microbiology and Molecular Biology Reviews : MMBR* **74**, 570--588 (2010).

2. Brázda, V., Laister, R.C., Jagelská, E.B. & Arrowsmith, C. Cruciform structures are a common DNA feature important for regulating biological processes. *BMC Molecular Biology* **12**, 1--16 (2011).
